# Strategy for treatment of stage IV human epidermal growth factor 2-positive gastric cancer: a case report

**DOI:** 10.1186/s13256-019-2001-3

**Published:** 2019-02-22

**Authors:** Masazumi Sakaguchi, Norihiro Shimoike, Shin Akagawa, Seiichiro Kanaya

**Affiliations:** Department of Surgery, Osaka Red Cross Hospital, 5-30 Fudegasakicho, Tennoji Ward, Osaka, 543-8555 Japan

**Keywords:** Stage IV gastric cancer, Human epidermal growth factor 2-positive gastric cancer, Conversion therapy, Trastuzumab

## Abstract

**Background:**

The prognosis of stage IV gastric cancer and human epidermal growth factor 2 (HER2)-positive gastric cancer is poor, although new drugs and regimens have been developed. We report a case of a patient with stage IV HER2-positive gastric cancer treated successfully by conversion therapy and trastuzumab.

**Case presentation:**

The patient was a 73-year-old Japanese man diagnosed as L, type 3, circ, T4aNxCy1P1M1, stage IV (the Japanese classification of gastric carcinoma). The patient was treated with docetaxel, cisplatin, and TS-1 (DCS regimen). After two courses of the regimen, peritoneal dissemination disappeared, and peritoneal lavage cytology revealed no tumor cells in the abdominal cavity. Subsequently, he underwent laparoscopic distal gastrectomy with D1+. Pathological findings were ypT2(MP), ypN2(3/15), ypP0, ypCY0, M0, ypstage II. He received TS-1 as an adjuvant chemotherapy, but he had peritoneal recurrence. The original gastric cancer was HER2-positive. We therefore treated him with TS-1 with trastuzumab. This regimen was quite effective and achieved a complete response. After complete response, we switched the patient to trastuzumab monotherapy. He had no evidence of recurrence for 6 years, 3 months after surgery.

**Conclusion:**

DCS regimen, R0 resection, and adjuvant chemotherapy with trastuzumab can be a powerful strategy for stage IV HER2-positive gastric cancer.

## Background

The prognosis of stage IV gastric cancer (GC) is poor. However, as new drugs and regimens have been developed, sometimes tumors have shown good response and they are converted from unresectable to resectable. Conversion therapy is defined as a surgical treatment aiming at R0 resection after chemotherapy for unresectable tumors. Conversion therapy can be one of the treatments for stage IV GC, but there are many problems, to be clear: proper preoperative and postoperative regimens, duration, timing of the operation, and so forth [1].

Human epidermal growth factor 2 (HER2) is a proto-oncogene encoded by *ERBB2* and associated with tumor cell proliferation and apoptosis [2, 3]. Some studies showed that HER2-positive GC is associated with poor outcomes [4–6]. It is treated with trastuzumab with chemotherapy based on the result of the ToGA trial [7]. According to the results of that trial, median overall survival was 13.8 months (95% confidence interval, 12–16) in patients with HER2-positive GC treated with trastuzumab with chemotherapy.

Few studies of conversion therapy against stage IV HER2-positive GC have been reported, owing to its low incidence. Patients with stage IV HER2-positive GC are usually treated with trastuzumab with chemotherapy; however, we treated our patient with another chemotherapy regimen without trastuzumab and performed conversion therapy. After peritoneal recurrence, trastuzumab was initiated, and a complete response (CR) was achieved; our strategy was successful. There were no reports about long-term survivors with stage IV HER2-positive GC, to our knowledge; therefore, we decided to report this suggestive case.

## Case presentation

**Fig. 1:**
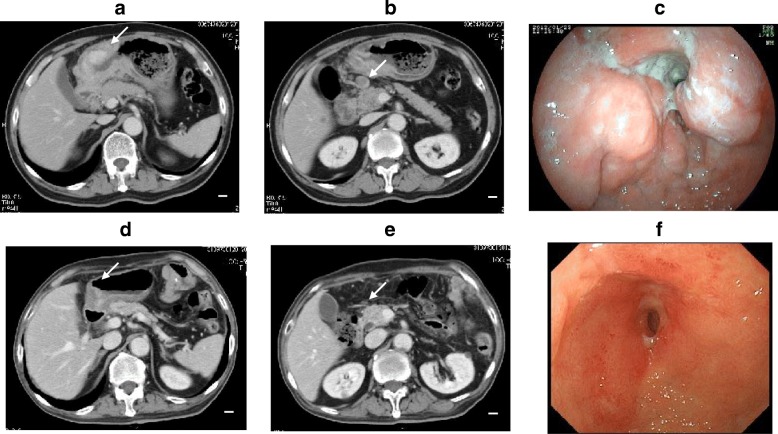
Computed tomography (CT) and esophagogastric fiber (EGF) findings at diagnosis and after two courses of docetaxel, cisplatin, and TS-1 chemotherapy. **a**, **b** Abdominal CT showed thickened wall of the antrum and a bulky lymph node (*arrows*). **c** EGF showed type 3 tumor in the antrum. **d**, **e** Thickened antrum wall was significantly improved, and the bulky lymph node was significantly shrunk (*arrows*). **f** Type 3 tumor was remarkably shrunk. Side bar = 1 cm

**Fig. 2:**
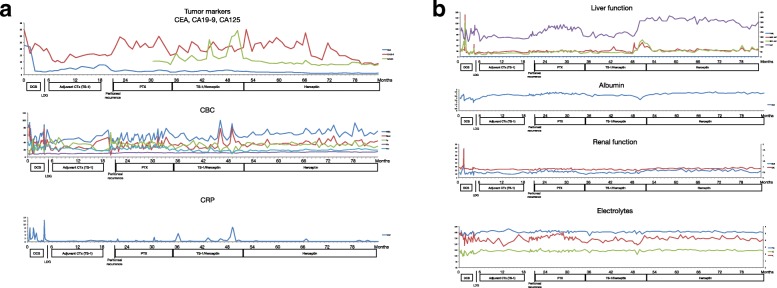
**a** Changes in tumor markers (CEA, CA19-9 and CA125), CBC, and CRP. **b** Changes in liver function, serum albumin, renal fanction and electrolytes. *CEA* Carcinoembryonic antigen (U/ml), *CA19-9* Cancer antigen 19-9 (U/ml), *CA125* Cancer antigen 125 (U/ml), *WBC* White blood cell count (× 100 per cubic milliliter), *Neut* Neutrophil (× 100 per cubic milliliter), *Hg* Hemoglobin (g/dl), *PLT* Platelet count (× 10,000 per cubic milliliter), *CRP* C-reactive protein (mg/L), *T-Bil* Total bilirubin (mg/dl), *AST* Aspartate transaminase (IU/L), *ALT* Alanine transaminase (IU/L), *ALP* Alkaline phosphatase (IU/L), *Alb* Albumin (g/dl), *BUN* Blood urea nitrogen (mg/dl), *CRE* Creatinine (mg/dl), *Na* Sodium (mEq/L), *Cl* Chloride (mEq/L), *P* Potassium (mEq/L)

**Fig. 3:**
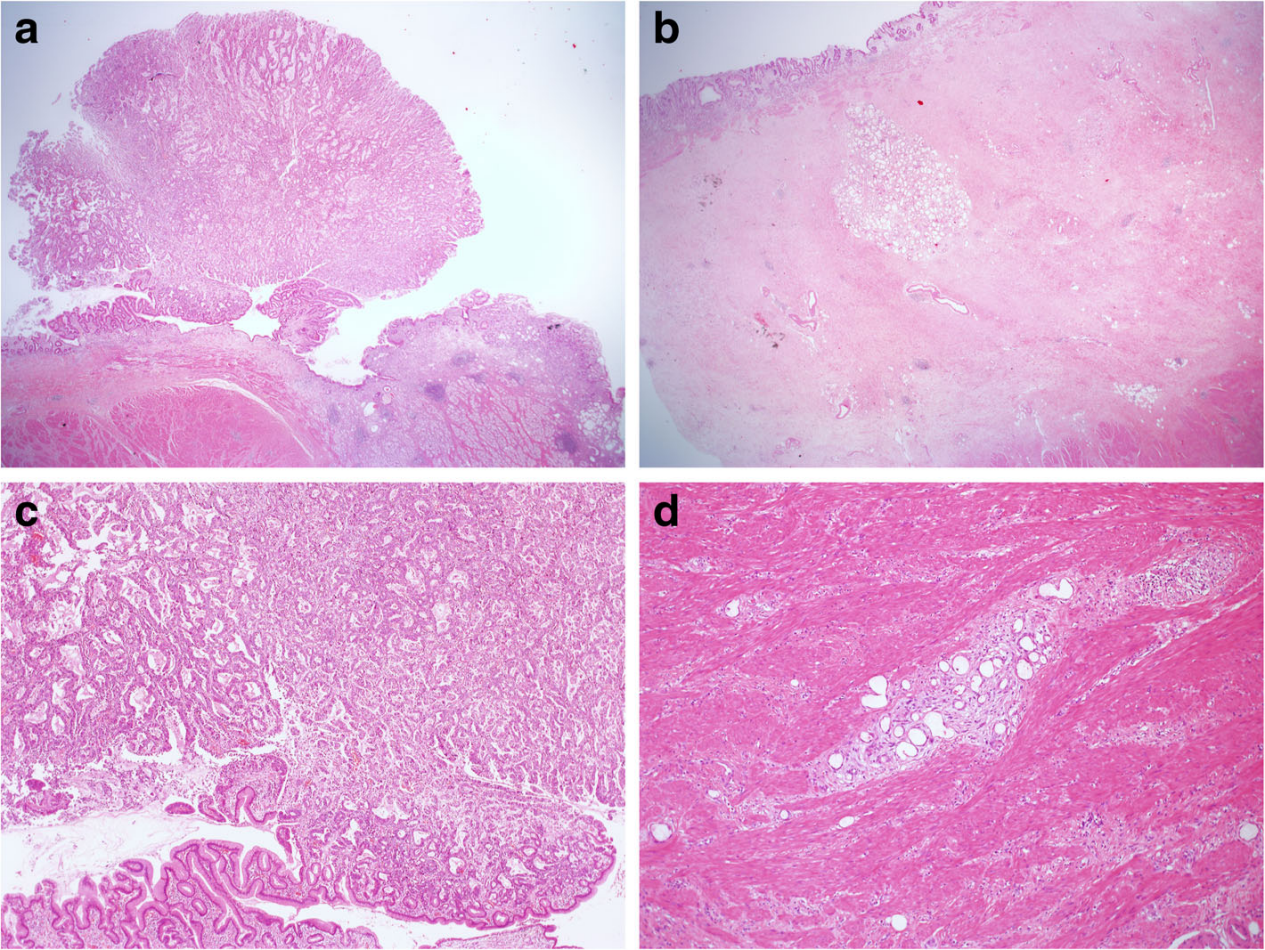
Pathological findings. **a** Microscopic view of the cancer (H&E stain, × 12.5). **b** Microscopic view showed fibrosis in subserosa in which the cancer cells seemed to have disappeared (H&E stain, × 12.5). **c** H&E stain showed massive number of cancer cells were present in subserosa (× 40). **d** Cancer cells were present in muscularis propria (H&E stain, × 100)

**Fig. 4:**
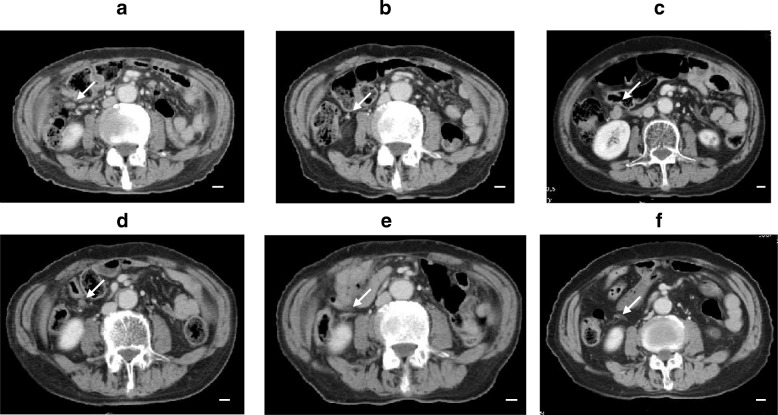
Trastuzumab is effective against HER2-positive gastric cancer. **a** Abdominal computed tomography (CT) showed a peritoneal nodule (*arrow*). **b** Paclitaxel was effective against peritoneal dissemination (*arrow*). **c** CT showed a new peritoneal nodule appeared *(arrow)*. **d**, **e** Peritoneal dissemination disappeared (*arrows*). **f** CT showed peritoneal dissemination was disappeared for 6 years and 3 months after surgery *(arrow)*

**Fig. 5:**
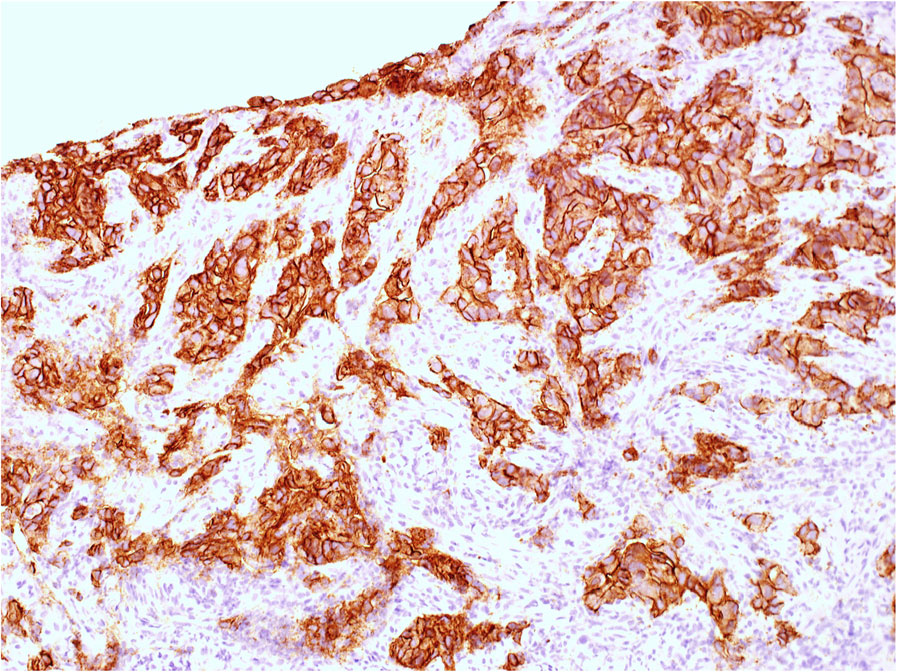
Immunostaining of the cancer. Immunostaining with HER2 showed the cancer cells were HER2-positive (× 200)

A 73-year-old Japanese man with a 2-month history of dysphasia and heartburn first presented to his local doctor and was later admitted to our hospital. He had difficulties in swallowing and eating; did not have melena, epigastralgia, or hematemesis; and had a history of hypertension and no known allergies. At the time of admission, he was taking at lansoprazole 15 mg/day and olmesartan medoxomil 10 mg/day. He did not drink alcohol but used to smoke 30 cigarettes per day for 45 years. His environmental and employment histories were unremarkable. His family history was remarkable for colon cancer in his father and lung cancer in his brother. On admission, his height was 161 cm, body weight was 56.5 kg, blood pressure was 126/62 mm Hg, pulse was 70 beats per minute, temperature was 36.9 °C, and oxygen saturation was 98% while he was breathing ambient air. His conjunctiva was not icteric but slightly anemic. On chest examination, his heart rhythm was regular with no murmur, and his lungs were clear to auscultation. His abdomen was soft, not distended, and not tender. A soft and movable mass was palpable around the epigastrium. The legs and feet showed no edema. Laboratory tests showed a creatinine level of 0.89 mg/dl, blood urea nitrogen level of 12.6 mg/dl, total bilirubin level of 0.3 mg/dl, aspartate transaminase level of 17 IU/L, and alanine transaminase level of 19 IU/L. The patient’s white blood cell count was 8930 per cubic milliliter, hemoglobin was 9.2 g/dl, and platelet count was 438,000 per cubic milliliter. An esophagogastric fiber (EGF) showed type 3 gastric carcinoma in the antrum. The tumor caused pyloric stenosis and invasion to the duodenum, so the patient was admitted to the hospital (Fig. 1a–c). Staging laparoscopy was performed to assess the extent of tumor spread, and laparoscopic bypass was performed. Staging laparoscopy revealed peritoneal dissemination, and peritoneal lavage cytology revealed tumor cells in the abdominal cavity. We diagnosed L, type 3, circ, cT4a(SE), cNx, pP1, pCY1, M0, stage IV (the Japanese classification of gastric carcinoma). The patient was initially treated with docetaxel 40 mg/m^2^ on day 1, cisplatin (CDDP) 60 mg/m^2^ on day 1, and TS-1 120 mg/day on days 1–14, followed by a 2-week recovery period (DCS regimen). Dexamethasone 9.9 mg and palonosetron 0.75 mg were administered on day 1, and dexamethasone 6.6 mg was administered on days 2 and 3 as premedication. The patient had grade 3 diarrhea (according to Common Terminology Criteria for Adverse Events criteria) after one course (Fig. 2a, b). Then TS-1 was reduced (100 mg). After two courses of the DCS regimen, EGF and computed tomography (CT) showed that the tumor had shrunk (Fig. 1c–e), and then staging laparoscopy was performed to evaluate a response. Peritoneal dissemination disappeared, and peritoneal lavage cytology revealed no tumor cells in the abdominal cavity. Then salvage operation, laparoscopic distal gastrectomy with D1+ dissection, was performed. Pathological findings were ypT2(MP), ypN2(3/15), ypP0, ypCY0, M0, ystage II (Fig. 3). TS-1100 mg/day on days 1–14, every 3 weeks was started as adjuvant chemotherapy. After 15 months, CT revealed multiple peritoneal nodules (Fig. 4a). They were highly suspected as a recurrence. Paclitaxel 80 mg/m^2^ on days 1, 8, and 15 was started as a second regimen. Dexamethasone 6.6 mg, famotidine 20 mg, and granisetron 3 mg were administered on days 1, 8, and 15 as premedication. This regimen achieved partial response (Fig. 4b), but its efficacy did not last. After 3 months, CT revealed progressive disease (Fig. 4c). The original gastric carcinoma was HER2-positive (Fig. 5). The patient’s Eastern Cooperative Oncology Group performance status was 2; his body weight was 50.7 kg; and he complained of appetite loss. We concluded that the patient could not tolerate doublet therapy. Therefore, TS-1100 mg on days 1–14 with Herceptin 6 mg/kg (Roche/Genentech, South San Francisco, CA, USA) on day 1 every 3 weeks was introduced. This regimen was substantially effective and achieved CR after 9 months based on CT findings (Fig. 4d, e). The patient had no adverse effects while receiving this regimen (Fig. 2a, b). Since then, the patient has been treated with only Herceptin 6 mg/kg every 3 weeks without any side effects, and no radiological findings of recurrence had yet occurred for 6 years, 7 months after surgery (Fig. 4f).

## Discussion

Our patient with stage IV HER2-positive GC was initially administered docetaxel, CDDP, and TS-1 (DCS regimen) to convert unresectable GC to resectable GC, despite trastuzumab with chemotherapy. The conversion therapy was successful. After peritoneal recurrence, trastuzumab with chemotherapy was introduced, which achieved a CR. Trastuzumab monotherapy was then started, and CR was maintained. This patient has remained alive without disease progression for 6 years, 7 months after surgery.

The number of conversion therapies for stage IV GC is increasing as the result of development of new drugs and regimens [8–12]. However, regimens as induction chemotherapy, timing of the operation, and regimens after surgery remain unclear. In this case, we selected DCS regimens as an induction chemotherapy and did not check HER2 status, because we planned conversion surgery and DCS showed a better response rate (RR, 81%) than that of chemotherapy with Herceptin (RR, 47%) [7, 13]. In addition to the difference in RR, Janjigian *et al*. reported loss of HER2 expression in patients with HER2-positive esophagogastric tumors treated with trastuzumab, resulting in resistance to trastuzumab [14]. Given these reports, trastuzumab with chemotherapy is not proper as an induction therapy. After two courses of chemotherapy, we performed staging laparoscopy to assess peritoneal dissemination and lavage cytology and confirmed no dissemination and no tumors in the abdominal cavity. Some studies showed that patients with stage IV GC who underwent R0 resection after chemotherapy had a better prognosis [8, 9, 12]. We therefore performed distal gastrectomy with D1+ resection aiming at R0 resection.

We started TS-1 as adjuvant chemotherapy. After 3 months, peritoneal recurrence was suspected, and we changed the regimen. Paclitaxel is known for good transition to the peritoneum, so we administered it to the patient. However, it was not effective. We started therapy with Herceptin because the patient’s original GC showed HercepTest 3+ (Dako, Carpinteria, CA, USA). The ToGA trial showed that six cycles of chemotherapy with trastuzumab and then trastuzumab monotherapy until disease progression was effective for HER2-positive GC. In this case, we selected only TS-1 for chemotherapy because some studies showed TS-1 was effective for peritoneal recurrence [15, 16]. We judged that the patient could not tolerate doublet therapy. TS-1 with trastuzumab was significantly effective, and the patient was able to tolerate this regimen, so we continued it until CR. After that, we continued trastuzumab monotherapy as maintenance therapy. To date, we have found no evidence of recurrence. To the best of our knowledge, there are no other reports of long-term survivors of stage IV HER2-positive GC who underwent conversion surgery.

Trastuzumab inhibits the HER2 signaling pathway in tumor cells expressing HER2 and induces antibody-dependent cell-mediated cytotoxicity through binding HER2-positive cancer cells [17]. The HER2 signal pathway is related to cancer cell proliferation and inhibition of apoptosis [18]. Trastuzumab monotherapy therefore has antitumor activity. However, more than half of patients with HER2-positive GC in the ToGA trial did not respond to trastuzumab [3]. Kataoka *et al.* reported that HER2 expression in gastroesophageal adenocarcinoma (GEA) is often heterogeneous [19]. Therefore, a predictive biomarker is needed to select patients who exhibit a good objective response to trastuzumab. Deguchi *et al*. showed that loss of phosphatase and tensin homolog (PTEN) contributed to trastuzumab resistance in a GEA cell line, and they concluded that PTEN loss can be a clinically valuable biomarker of resistance to trastuzumab [20]. In our patient, trastuzumab monotherapy could suppress peritoneal recurrence after surgery. Therefore, it is possible that PTEN was intact in our patient, although we did not assess it.

## Conclusion

In summary, for patients with stage IV HER2-posisitve GC, the strategy should be to include a DCS regimen as induction therapy and to perform conversion therapy. The adjuvant chemotherapy is TS-1 for 1 year. If there is a recurrence, trastuzumab with chemotherapy is introduced and is switched to trastuzumab monotherapy after six cycles. Trastuzumab monotherapy is continued until disease progression. Owing to its low incidence, there is no established regimen for conversion therapy against stage IV HER2-positive GC. On the basis of this experience, our strategy can be a powerful treatment for stage IV HER2-positive GC.

## Abbreviations

CR: Complete response
CT: Computed tomography
EGF: Esophagogastric fiber
GC: Gastric cancer
GEA: Gastroesophageal adenocarcinoma
HER2: Human epidermal growth factor 2
PTEN: Phosphatase and tensin homolog
RR: Response rate
